# Gender differences in the effect of rtms with the H7-coil on physical and social anhedonia in schizophrenia spectrum disorder; a randomized, sham-controlled trial

**DOI:** 10.1192/j.eurpsy.2024.509

**Published:** 2024-08-27

**Authors:** K. Matić, I. Šimunović Filipčić, I. Orgulan, Ž. Milovac, Ž. Bajić, I. Filipčić

**Affiliations:** ^1^Psychiatric Clinic Sveti Ivan; ^2^Department of psychiatry and psychological medicine, University Hospital Centre Zagreb, Zagreb; ^3^Facutly of Dental Medicine, Josip Juraj Strossmayer University of Osijek, Osijek, Croatia

## Abstract

**Introduction:**

Studies of differences in the incidence and severity of physical and social anhedonia between women and men diagnosed with schizophrenia spectrum disorder (SSD) are often inconsistent, and gender differences in treatment response have not been well studied. Hormonal factors, such as those related to the menstrual cycle, pregnancy or menopause, as well as social and cultural patterns and roles, may influence treatment response. The incidence of affective or stress-related psychiatric comorbidities may be gender-specific, which could also complicate the treatment of anhedonia and other negative symptoms of SSD. Finally, there is no evidence of sufficient quality on gender differences in the effects of rTMS, but the results are intriguing and point to the need for further research.

**Objectives:**

To investigate gender differences in the effect of rTMS with the H7-coil on physical and social anhedonia in patients diagnosed with SSD with dominant negative symptoms.

**Methods:**

We conducted a randomized, sham-controlled trial during 2000-2023 in the population of patients diagnosed with SSD with primary negative symptoms defined as PANSS negative symptoms subscale score > 24, and PANSS positive symptoms subscale score < 20. The intervention was HF rTMS H7 coil (Brainsway Ltd. Jerusalem, Israel) once daily for 20 days applied to the prefrontal cortex (mPFC and ACC) at 100% motor threshold with a frequency of 18 Hz, and total of 39600 pulses. The outcomes were Physical and Social Anhedonia Scales (PAS, and SAS). We controlled for the large number of relevant covariates.

**Results:**

We randomized 49 men and 29 women of similar age. The effect on physical anhedonia was statistically significant in women (b = 9.04; p = 0.016), but not in men (b = 2.87; p = 0.272). The effect on social anhedonia was similar, but the difference was smaller (for men b = 3.71; p = 0.082; for women b = 5.42; p = 0.043). However, the Wald test showed no statistically significant differences between the beta coefficients for women and men.

**Conclusions:**

Based on this study, it is not possible to make valid and reliable conclusions about the existence of gender differences in the effects of rTMS treatment of anhedonia with the H7 coil. However, it is possible to claim that the treatment of anhedonia with this protocol is effective in women.

**Disclosure of Interest:**

None Declared

